# Identification of Possible Virulence Marker from *Campylobacter jejuni* Isolates

**DOI:** 10.3201/eid2006.130635

**Published:** 2014-06

**Authors:** James W. Harrison, Tran Thi Ngoc Dung, Fariha Siddiqui, Sunee Korbrisate, Habib Bukhari, My Phan Vu Tra, Nguyen Van Minh Hoang, Juan Carrique-Mas, Juliet Bryant, James I. Campbell, David J. Studholme, Brendan W. Wren, Stephen Baker, Richard W. Titball, Olivia L. Champion

**Affiliations:** University of Exeter, Exeter, UK (J.W. Harrison, D.J. Studholme, R.W. Titball, O.L. Champion);; The Hospital for Tropical Diseases, Oxford University Clinical Research Unit, Ho Chi Minh City, Vietnam (T.T.N. Dung, M.P.V. Tra, N.V.M. Hoang, J. Carrique-Mas, J. Bryant, J.I. Campbell, S. Baker);; Comsats University, Islamabad, Pakistan (F. Siddiqui, H. Bukhari);; Mahidol University, Bangkok, Thailand (S. Korbrisate);; University of Oxford, Oxford, UK (J. Carrique-Mas, J. Bryant, J.I. Campbell, S. Baker);; London School of Hygiene and Tropical Medicine, London, UK (B.W. Wren)

**Keywords:** Campylobacter jejuni, Asia, chicken, type-six secretion system, type-6 secretion system, food security, enteric infections, diarrhea, Thailand, virulence, bacteria

## Abstract

A novel protein translocation system, the type-6 secretion system (T6SS), may play a role in virulence of *Campylobacter jejuni*. We investigated 181 *C. jejuni* isolates from humans, chickens, and environmental sources in Vietnam, Thailand, Pakistan, and the United Kingdom for T6SS. The marker was most prevalent in human and chicken isolates from Vietnam.

*Campylobacter* species are the principal bacterial cause of human foodborne enterocolitis worldwide (*1*). Despite the global significance of *C. jejuni* as a leading cause of diarrheal disease (*2*), the mechanisms of pathogenesis of *C. jejuni* are not well understood. Research on *Campylobacter* epidemiology has largely been conducted in high-income countries and therefore may not be representative of global patterns. 

Recently, a novel class of protein translocation system was identified in gram-negative bacteria. This system, named the type-6 secretion system (T6SS), has been found to play roles in pathogen–pathogen and host–pathogen interactions and has a major effect on virulence in a range of pathogens, including *Vibrio cholerae* (*3**–**6*) (reviewed in *7*,*8*). A functional T6SS was recently identified in *C. jejuni* (*9*,*10*) and found to have several roles in virulence, influencing cell adhesion, cytotoxicity toward erythrocytes, and colonization of mice (*9*,*10*). However, it is unknown whether T6SS changes the effects of these pathogens in human infection. 

In this study, we aimed to determine whether presence of T6SS in *C. jejuni* is potentially a marker associated with more severe human disease. Moreover, because human infection with *C. jejuni* is often associated with contact with poultry, we investigated whether poultry harbor *C. jejuni* that possess T6SS.

## The Study

**Figure 1:**
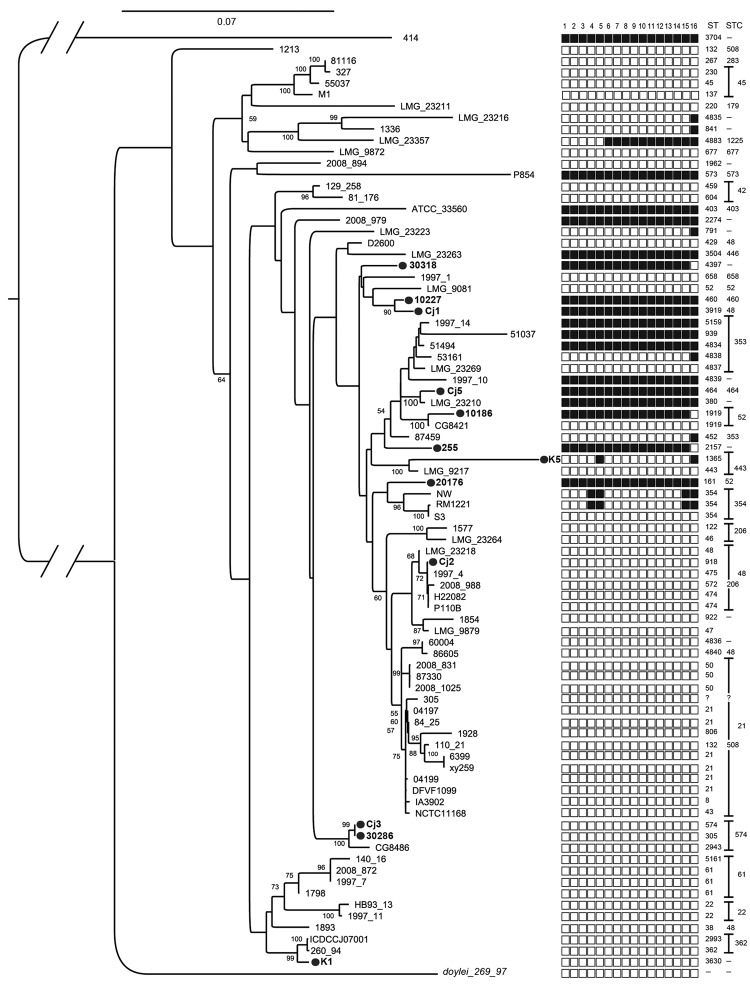
Distribution of the type-six secretion system (T6SS) marker across the phylogenetic diversity of *Campylobacter jejuni* strains, as determined by multilocus sequence analysis. We generated a maximum-likelihood tree from concatenated nucleotide alignments of 31 housekeeping genes; nucleotide sequences were aligned by using MUSCLE (www.drive5.com/muscle) and masked by using GBLOCKS (http://molevol.cmima.csic.es/castresana/Gblocks.html). Maximum-likelihood analysis was done by using the GTR model in PhyML (http://code.google.com/p/phyml/). Numbers on nodes denote bootstrap values (1,000 bootstrap replicates); values <50 are not shown. Black circles indicate strains whose genomes were sequenced in this study (GenBank accession nos. AUUQ00000000, AUUP00000000, AUUO00000000, AUUN00000000, AUUM00000000, AUUL00000000, AUUK00000000, AUUJ00000000, AUUI00000000, ARWS00000000, AUUH00000000, AUUG00000000). We inferred the presence/absence of each of the T6SS genes on the basis of TBLASTN (http://blast.ncbi.nlm.nih.gov/Blast.cgi?PROGRAM=blastn&PAGE_TYPE=BlastSearch) searches against the predicted proteins sequences from *C. jejuni* strain 414 (National Center for Biotechnology Information reference sequence no. NZ_CM000855). Presence or absence of each gene is indicated by a black or white square, respectively, for each strain: column 1, *hcp*; column 2, *icmF*_1; column 3, *icmF*_2; column 4, *vasK*; column 5, *FHA*; column 6, *vasF*; column 7, *vasE*; column 8, *vasD*; column 9, *impA*; column 10, *impD*; column 11, *impC*; columns 12 and 13, conserved hypotheticals; column 14, *vasA*; column 15, *vasB*; column 16, *vgrg*. The sequence type (ST) and ST complex (STC) columns represent global multilocus sequence types as described by the Oxford multilocus sequence typing scheme (http://pubmlst.org). ?, unknown ST; –, isolate could not be allocated to a specific ST or STC. Further details of the isolates are provided in Technical Appendix Table 2.

To partially address bias toward study of *C. jejuni* strains from high-income countries and the under-representation of strains from Asia in previous studies, we previously sequenced the genomes of 12 clinical isolates of *C. jejuni* from Asia: 4 from Thailand, 3 from Pakistan, and 5 from Vietnam (J. Harrison, unpub. data; Figure 1). We noted that 8 (67%) of these isolates possessed a cluster of genes homologous to the recently described T6SS (Figure 1). This finding was in contrast to findings regarding previously sequenced *C. jejuni* genomes; only 10 (14%) of 71 previously sequenced *C. jejuni* strains possessed an apparently intact T6SS gene cluster (Figure 1; full listing of genomes is in Technical Appendix Table 1). Several other strains from our study and previously sequenced strains contained >1 T6SS genes but not a complete T6SS cluster. Figure 1 shows the presence and absence of each T6SS gene in each available genome sequence (J. Harrison, unpub. data) and the previously sequenced strains. A nonrandom distribution of T6SS can be seen across the phylogenetic diversity of *C. jejuni*; T6SS is limited to certain clades, and degeneration of the T6SS gene cluster apparently occurs in parallel within several of those clades (Figure 1).

Our genome sequencing analysis indicated that strains possessing a complete T6SS cluster could be distinguished by the presence of the *hcp* gene (Figure 1) (*9*,*10*). Therefore, we used *hcp* as a proxy for determining the presence of a functional T6SS in 181 *C. jejuni* isolates from chickens, humans, and environmental sources (collections of the Oxford University Clinical Research Unit and the University of Exeter; online Technical Appendix Table 2). We designed and used a multiplex PCR (Technical Appendix Table 3) to screen for the presence of *hcp* in these isolates; the conserved *C. jejuni* housekeeping gene, *gltA*, was used as a positive control.

Of the 181 isolates, 28 originated from chickens in the United Kingdom and 21 from chickens in Vietnam. The *hcp* gene was found significantly more often in isolates from Vietnam (15 [71.4%] isolates) than in those from the United Kingdom (1 [3.5%] isolate) (p<0.01 by 2-sample *Z*-test; Technical Appendix Figure 1). An additional 38 of the isolates were from humans in the United Kingdom and 33 from humans in Vietnam; again, the *hcp* gene was significantly more prevalent in isolates from Vietnam (20 [60.6%] isolates) than those from the United Kingdom (1 [2.6%] isolate) (p<0.01 by 2-sample *Z*-test; Technical Appendix Figure 2).

**Figure 2:**
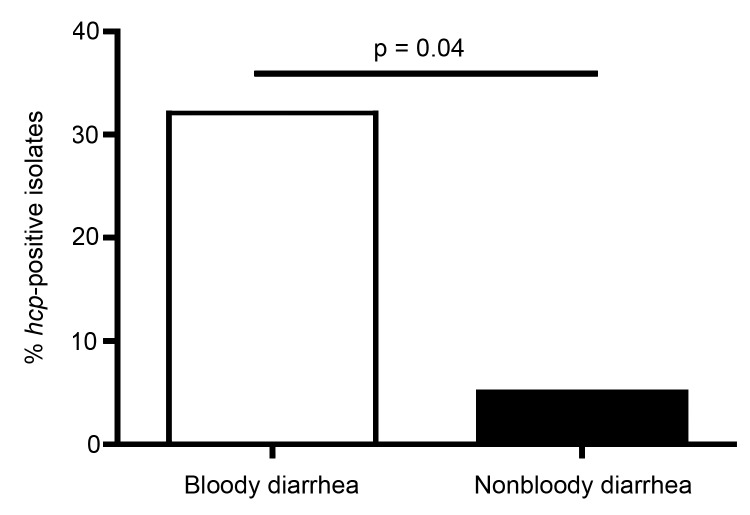
Percentage of *hcp*-positive *Campylobacter jejuni* strains isolated from patients in Vietnam who had bloody diarrhea and nonbloody diarrhea. Patients who were hospitalized because of *C. jejuni* infection were scored for the presence of bloody diarrhea or nonbloody diarrhea, and presence of the *hcp* type-six secretion system (T6SS) marker in strains isolated from the patients was determined. Of patients with bloody diarrhea, 32% were infected with *hcp*-positive strains; of patients with nonbloody diarrhea, 5% were infected with *hcp*-positive strains.

We also found that patients infected with *hcp*-positive *C. jejuni* experienced bloody diarrhea more commonly than those infected with *hcp*-negative *C. jejuni*. For the 36 isolates for which detailed clinical data on patients were available, 6 (31.6%) of 19 patients in Vietnam who were infected with *hcp*-positive *C. jejuni* had bloody diarrhea, compared with 1 (5.9%) of 17 patients infected with *hcp*-negative *C. jejuni* (p<0.05 by 2-sample *Z*-test) (Figure 2). These results suggest a potential correlation between T6SS and bloody diarrhea, a serious clinical manifestation of the infection that results in higher rates of hospitalization and greater need for treatment with antimicrobial drugs (*11*). Moreover, *Campylobacter*-related septicemia developed in the 1 patient in the United Kingdom who was infected with a T6SS-positive strain (*11*). These data suggest that infection with the *C. jejuni* T6SS genotypic strains is associated with more severe disease. However, for sample bias to be ruled out, a comprehensive study is required in which the prevalence of T6SS is measured in *C. jejuni* samples from patients with mild and severe forms of infection.

We found a number of *C. jejuni* strains from humans and poultry that possessed the T6SS cluster, although some strains showed a slightly modified gene order (Technical Appendix Table 1 and Figure 3). However, most (61 [85.9%] of 71) of the previously sequenced *C. jejuni* isolates lacked a complete T6SS gene cluster (Figure 1); this finding might explain why T6SS was not discovered in *C. jeuni* sooner. Conversely, our PCR-based study frequently identified the *hcp* marker in isolates from Thailand, Pakistan, and Vietnam (Table). We cannot be certain that all of the isolates with the *hcp* marker possessed a complete and functional T6SS gene cluster, but the *hcp* gene is consistently associated with the presence of a complete T6SS cluster in all available sequenced *C. jejuni* genomes (Figure 1). This correlation lends confidence to the use of *hcp* as a proxy.

**Table:** Overview of *Campylobacter jejuni* strains containing type-six secretion system genetic marker *hcp*, by country and isolate source

Isolate source	No. *hcp*-positive strains/total no. strains (%)				
	United Kingdom	Vietnam	Pakistan	Thailand	Total
Human	1/38 (2.6)	20/33 (60.6)	2/13 (15.4)	1/3 (33.3)	24/87 (27.6)
Chicken	1/28 (3.9)	15/21 (71.4)	1/2 (50)	0	17/51 (33.3)
Other	5/26 (19.2)	1/14 (7.1)	1/3 (33.3)	0	7/43 (16.3)
Total	7/92 (7.6)	36/68 (54.4)	4/18 22.2)	1/3 (33.3)	48/181 (26.5)

Poultry are a well-documented reservoir of human *Campylobacter* infection (*12*). We found that *Campylobacter* strains harboring the *hcp* marker were significantly associated with chickens in Asia. Large numbers of poultry are imported into North America and Europe from low-income countries, including Thailand (*13*). This process could introduce T6SS-positive *Campylobacter* genotypes into the food chains of the importing countries, posing a potential emerging threat to public health.

## Conclusions

Our results suggest that the T6SS may be more prevalent in *C. jejuni* in Vietnam, Pakistan, and Thailand than in the United Kingdom. Furthermore, our results suggest that *hcp* may be a marker associated with severe human disease caused by *C. jejuni* infection in Vietnam, although there is no evidence that the association is causal. Chickens imported from these countries could be a source of *hcp-*positive strains and may have the potential to cause severe human infection.

Technical Appendix Supplementary methods and primers used to identify *hcp* and *gltA* genes in *Campylobacter jejuni*, lists of *C. jejuni* strains included in analyses, prevalence of type-six secretion system (T6SS) genetic marker *hcp* in *C. jejuni* isolated from chickens and humans in Vietnam and the UK, and comparison of the gene orders in the T6SS gene clusters found in *C. jejuni*.
