# Supplementary material for: Identification of Possible Virulence Marker from Campylobacter jejuni Isolates
Source: Emerg Infect Dis. 2014 Jun;20(6):1026–9. doi: 10.3201/eid2006.130635 (PMC4036754; doi:10.3201/eid2006.130635)
Supplement: Technical Appendix — Supplementary methods and primers used to identify hcp and gltA genes in Campylobacter jejuni, lists of C. jejuni strains included in analyses, prevalence of type-six secretion system (T6SS) genetic marker hcp in C. jejuni isolated from chickens and humans in Vietnam and the UK, and comparison of the gene orders in the T6SS gene clusters found in C. jejuni. [file 13-0635-Techapp-s1.pdf]

Article DOI: http://dx.doi.org/10.3201/eid2006.130635

Identification of Possible Virulence Marker from *Campylobacter jejuni* Isolates

Technical Appendix

Supplementary Methods

Multiplex PCR analysis was used for identification of the *hcp* and *gltA* genes. Oligonucleotide primers were designed using the Cj1 sequence (Technical Appendix Table 3). PCRs were carried out in a volume of 25 µl. This reaction consisted of 0.2 mM of each dNTP (Invitrogen), 0.25 unit of TaqDNA polymerase (BioLabs), 0.4 µM of the downstream and upstream *hcp* primer, and 1–100 ng of template DNA. PCR was carried out with a DNA Engine Peltier Thermal Cycler (Bio Rad). Primers for internal control gene *gltA* were added at a concentration of 0.05 µM in each of the reaction mixtures to check the fidelity of the PCR. A reaction mixture without template DNA was used as the negative control. DNA extracted from *C. jejuni* clinical isolate Cj1 was used a positive control for amplification of primers. PCR products were analyzed on 1.5% agarose gel stained with SYBR Safe DNA stain.

Technical Appendix Table 1. List of *C. jejuni* strains included in MLSA analysis.

strain name	source	country of origin	T6SS	Genome status	Ref	Hcp +ve
305	Turkey	Germany	negative	draft	[1]	
327	Turkey	Unknown	negative	draft	[2]	
414	Bank Vole	Unknown	positive	complete	[3]	yes
1213	Cow	USA	negative	draft		
1336	Bird	Unknown	positive	complete	[3]	
1577	Cow	USA	negative	draft		
1798	Cow	USA	negative	draft		
1854	Cow	USA	negative	draft		
1893	Cow	USA	negative	draft		
1928	Cow	USA	negative	draft		
04197	Unknown	Unknown	negative	draft		
04199	Unknown	Unknown	negative	draft		
6399	Unknown	Unknown	negative	draft		
51037	chicken	USA	positive	draft		yes
51494	chicken	USA	positive	draft		yes
53161	chicken	USA	positive	draft		
60004	chicken	USA	negative	draft		
81116	human	Unknown	negative	complete	[4]	
86605	chicken	USA	negative	draft		
87330	chicken	USA	negative	draft		
87459	chicken	USA	positive	draft		
110_21	Unknown	USA	negative	draft		
129_258	Cow	USA	negative	draft		
140_16	Cow	USA	negative	draft		

strain name	source	country of origin	T6SS	Genome status	Ref	Hcp +ve
1997_1	Human	USA	negative	draft		
1997_10	Human	USA	positive	draft		yes
1997_11	Human	USA	negative	draft		
1997_14	Human	USA	positive	draft		yes
1997_4	Human	USA	negative	draft		
1997_7	Human	USA	negative	draft		
2008_1025	Human	France	negative	draft		
2008_831	Human	France	negative	draft		
2008_872	Human	France	negative	draft		
2008_894	Human	France	negative	draft		
2008_979	Human	France	positive	draft		yes
2008_988	Human	France	negative	draft		
260_94	Human	S. Africa	negative	draft		
81_176	Human	Unknown	negative	Complete	[5]	
84_25	Human	Unknown	negative	Complete		
ATCC_33560	Cow	Brussels	positive	draft		yes
CG8421	Human	Thailand	negative	draft	[6]	
CG8486	Human	Thailand	negative	draft	[7]	
D2600	Human	USA	negative	draft	[8]	
DFVF1099	chicken	Unknown	negative	draft	[1]	
H22082	Human	New Zealand	negative	draft	[1]	
HB93_13	Human	China	negative	draft	[9]	
IA3902	Sheep	USA	negative	Complete	[10]	
ICDCCJ07001	Human	China	negative	draft	[11]	
LMG_23210	chicken	Belgium	positive	draft		Yes
LMG_23211	chicken	Belgium	negative	draft		
LMG_23216	chicken	Belgium	positive	draft		
LMG_23218	chicken	Belgium	negative	draft		
LMG_23223	chicken	Belgium	positive	draft		
LMG_23263	chicken	Bosnia and Herzegovina	positive	draft		yes
LMG_23264	Human	Slovenia	negative	draft		
LMG_23269	chicken	Belgium	negative	draft		
LMG_23357	water	netherlands	positive	draft		
LMG_9081	human	USA	negative	draft		
LMG_9217	Human	Belgium	negative	draft		
LMG_9872	Human	Sweden	negative	draft		
LMG_9879	Human	Canada	negative	draft		
M1	Human/poultry	Unknown	negative	complete	[12]	
NCTC11168	Human	Unknown	negative	complete	[13]	
NW	Human	USA	positive	draft	[8]	
P110B	chicken	New Zealand	negative	draft	[14]	
P854	chicken	UK	positive	draft		yes
RM1221	Unknown	Unknown	positive	complete	[15]	
S3	poultry	Unknown	negative	complete	[16]	
doylei 269 97	Human	Unknown	negative	complete		
xy259	Unknown	Unknown	negative	draft		
55037	chicken	USA	negative	draft		

Technical Appendix Table 2. List of 181 *C. jejuni* strains analyzed in this study

strain name	source	country of origin	T6SS	Genome status	Ref	Strain source
28766	Beach	UK	negative			This study
KSCattle8	Cattle	UK	negative			This study
11974	human	UK	negative			This study
13305	human	UK	negative			This study
11919	human	UK	negative			This study
30280	human	UK	negative			This study
11818	human	UK	negative			This study
12241	human	UK	negative			This study
99/188	human	UK	negative			This study
99/197	human	UK	negative			This study
99/97	human	UK	negative			This study
0 1/ 43	human	UK	negative			This study

strain name	source	country of origin	T6SS	Genome status	Ref	Strain source
99/189	human	UK	negative			This study
99/216	human	UK	negative			This study
94/229	human	UK	negative			This study
99/212	human	UK	negative			This study
BB1267	human	UK	negative			This study
31467	human	UK	negative			This study
31484	human	UK	negative			This study
32799	human	UK	negative			This study
31485	human	UK	negative			This study
33084	human	UK	positive			This study
93/372	human	UK	negative			This study
32787	human	UK	negative			This study
44119	human	UK	negative			This study
47693	human	UK	negative			This study
33106	human	UK	negative			This study
34007	human	UK	negative			This study
Hi40980306	human	UK	negative			This study
90843	human	UK	negative			This study
Hi40500471	human	UK	negative			This study
Hi40620306	human	UK	negative			This study
BB1267	human	UK	negative			This study
Hi81266	human	UK	negative			This study
Hi80586	human	UK	negative			This study
Hi80547	human	UK	negative			This study
Hi81006	human	UK	negative			This study
KSSAPSM6	human	UK	negative			This study
Hi81214	human	UK	negative			This study
KSSHPSM4	human	UK	negative			This study
99/118	Cow	UK	negative			This study
99/201	Cow	UK	negative			This study
99/202	Cow	UK	negative			This study
C0599 3095	Cow	UK	negative			This study
C085 40995	Cow	UK	negative			This study
1182 ENV	Env	UK	negative			This study
PS304	Pig	UK	negative			This study
PS623	Pig	UK	positive			This study
PS762	Pig	UK	negative			This study
PS830	Pig	UK	negative			This study
PS838	Pig	UK	negative			This study
PS843	Pig	UK	positive			This study
PS849	Pig	UK	positive			This study
PS852	Pig	UK	positive			This study
PS857	Pig	UK	positive			This study
C120/2	Poultry	UK	negative			This study
C132/1	Poultry	UK	negative			This study
D2/T/80	Poultry	UK	negative			This study
PS55491	Poultry	UK	positive			This study
A83515A	Poultry	UK	negative			This study
A1CF12	Poultry	UK	negative			This study
D502009A	Poultry	UK	negative			This study
C3/T2/8	Poultry	UK	negative			This study
D2/27B	Poultry	UK	negative			This study
C3/T/25	Poultry	UK	negative			This study
EX1286	Poultry	UK	negative			This study
MB1	Poultry	UK	negative			This study
MB2	Poultry	UK	negative			This study
MB3	Poultry	UK	negative			This study
MB4	Poultry	UK	negative			This study
MB5	Poultry	UK	negative			This study
MB6	Poultry	UK	negative			This study
MB7	Poultry	UK	negative			This study
MB8	Poultry	UK	negative			This study
MB9	Poultry	UK	negative			This study
MB10	Poultry	UK	negative			This study

strain name	source	country of origin	T6SS	Genome status	Ref	Strain source
MB12	Poultry	UK	negative			This study
MB13	Poultry	UK	negative			This study
MB14	Poultry	UK	negative			This study
MB15	Poultry	UK	negative			This study
MB16	Poultry	UK	negative			This study
MB17	Poultry	UK	negative			This study
MB18	Poultry	UK	negative			This study
S2160509901	Sheep	UK	negative			This study
S390209903	Sheep	UK	negative			This study
S1200409904	Sheep	UK	negative			This study
S8704099	Sheep	UK	negative			This study
S3720509904	Sheep	UK	negative			This study
S3790809901	Sheep	UK	negative			This study
S43503099	Sheep	UK	negative			This study
S4990109905	Sheep	UK	negative			This study
S58503099	Sheep	UK	negative			This study
Cj 54	Camel	Pakistan	negative			This study
N2	human	Pakistan	negative			This study
AKRH011	human	Pakistan	negative			This study
702	human	Pakistan	negative			This study
Y25	human	Pakistan	negative			This study
2960HF	human	Pakistan	negative			This study
712	human	Pakistan	negative			This study
K1	human	Pakistan	negative	draft		This study
K2	human	Pakistan	positive			This study
K4	human	Pakistan	negative			This study
K5	human	Pakistan	negative	draft		This study
K6	human	Pakistan	negative			This study
K7	human	Pakistan	negative			This study
K8	human	Pakistan	positive			This study
80	Poultry	Pakistan	negative			This study
255	Poultry	Pakistan	positive	draft		This study
Cj245	waste water	Pakistan	negative			This study
Cj 236	waste water	Pakistan	positive			This study
Cj1	human	Thailand	positive	draft		This study
Cj2	human	Thailand	negative	draft		This study
Cj3	human	Thailand	negative	draft		This study
Cj5	human	Thailand	positive	draft		This study
20157	human	Vietnam	positive			This study
30286	human	Vietnam	positive	draft		This study
30261	human	Vietnam	positive			This study
10227	human	Vietnam	positive	draft		This study
20160	human	Vietnam	negative			This study
30106	human	Vietnam	negative			This study
20288	human	Vietnam	negative			This study
30311	human	Vietnam	positive			This study
20283	human	Vietnam	positive			This study
10186	human	Vietnam	positive	draft		This study
20176	human	Vietnam	positive	draft		This study
20231	human	Vietnam	positive			This study
20301	human	Vietnam	positive			This study
30318	human	Vietnam	positive	draft		This study
20321	human	Vietnam	positive			This study
20332	human	Vietnam	negative			This study
30355	human	Vietnam	positive			This study
20319	human	Vietnam	positive			This study
20137	human	Vietnam	positive			This study
30391	human	Vietnam	negative			This study
30396	human	Vietnam	negative			This study
10275	human	Vietnam	negative			This study
20227	human	Vietnam	positive			This study
30446	human	Vietnam	positive			This study
20127	human	Vietnam	positive			This study

strain name	source	country of origin	T6SS	Genome status	Ref	Strain source
20396	human	Vietnam	negative			This study
10126	human	Vietnam	positive			This study
20084	human	Vietnam	negative			This study
30431	human	Vietnam	negative			This study
30146	human	Vietnam	negative			This study
10070	human	Vietnam	negative			This study
10152	human	Vietnam	negative			This study
20245	human	Vietnam	positive			This study
71V103	Duck	Vietnam	negative			This study
71V42	Duck	Vietnam	negative			This study
71V489	Duck	Vietnam	negative			This study
71V151	Duck	Vietnam	negative			This study
71V135	Duck	Vietnam	negative			This study
71V445	Duck	Vietnam	negative			This study
71V484	Duck	Vietnam	negative			This study
71V420	Duck	Vietnam	negative			This study
71V409	Duck	Vietnam	negative			This study
71V397	Duck	Vietnam	negative			This study
71V49	Duck	Vietnam	negative			This study
71V69	Duck	Vietnam	negative			This study
72H57	Pig	Vietnam	negative			This study
71V110	Duck	Vietnam	positive			This study
71G139	Chicken	Vietnam	negative			This study
71G142	Chicken	Vietnam	positive			This study
71G356	Chicken	Vietnam	positive			This study
71G570	Chicken	Vietnam	positive			This study
71G784	Chicken	Vietnam	positive			This study
71G998	Chicken	Vietnam	positive			This study
71G1212	Chicken	Vietnam	positive			This study
71G1426	Chicken	Vietnam	positive			This study
71G1640	Chicken	Vietnam	positive			This study
71G1854	Chicken	Vietnam	positive			This study
71G2068	Chicken	Vietnam	positive			This study
71G2282	Chicken	Vietnam	positive			This study
71G326	Chicken	Vietnam	negative			This study
71G143	Chicken	Vietnam	positive			This study
71G329	Chicken	Vietnam	negative			This study
71G125	Chicken	Vietnam	positive			This study
71G124	Chicken	Vietnam	negative			This study
71G90	Chicken	Vietnam	positive			This study
71G30	Chicken	Vietnam	positive			This study
71G43	Chicken	Vietnam	negative			This study
72G117	Chicken	Vietnam	negative			This study

*Boldface indicates strains used in both the MLSA analysis and molecular epidemiology.

Technical Appendix Table 3. Primers used to PCR amplify the *hcp* and *gltA* genes

Primers (for target genes)	Primer sequence (5′→3′)	Predicted amplicon size	Tm	Reference
gltA F Cj	GCCCAAAGCCCATCAAGCGGA	142 bp	60	This study
gltA F Cj	GCGCTTTGGGGTCATGCACA		58	This study
Hcp F	CAAGCGGTGCATCTACTGAA	463 bp	60	This study
Hcp R	TAAGCTTTGCCCTCTCTCCA		60	This study

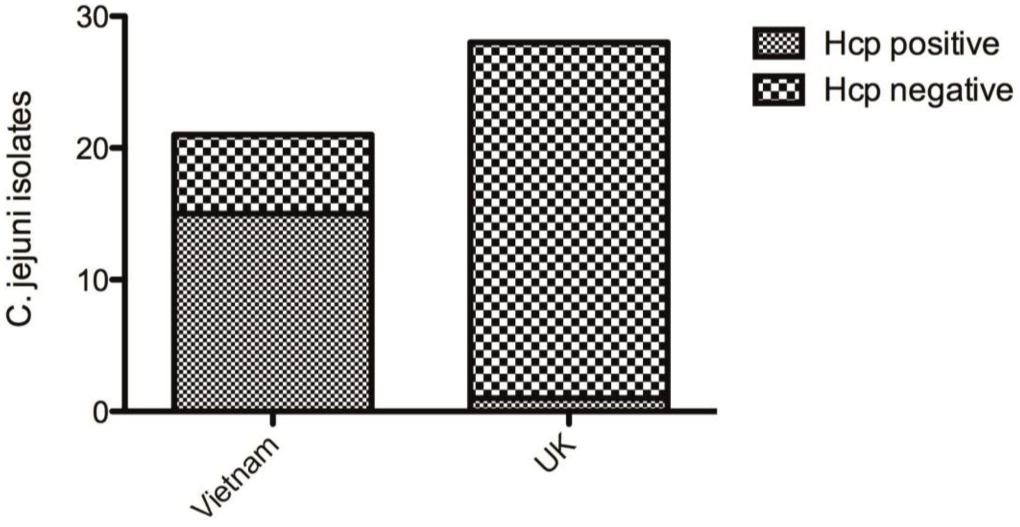

Technical Appendix Figure 1. Prevalence of T6SS genetic marker *hcp* in *Campylobacter jejuni* isolated from chickens in Vietnam and the UK. Multiplex PCR was performed on genomic DNA purified from *C. jejuni* that were isolated from chickens from Vietnam and the UK. Conserved T6SS gene *hcp* was used as a marker for a complete T6SS cluster. The conserved housekeeping gene *gltA* was used as a positive control.

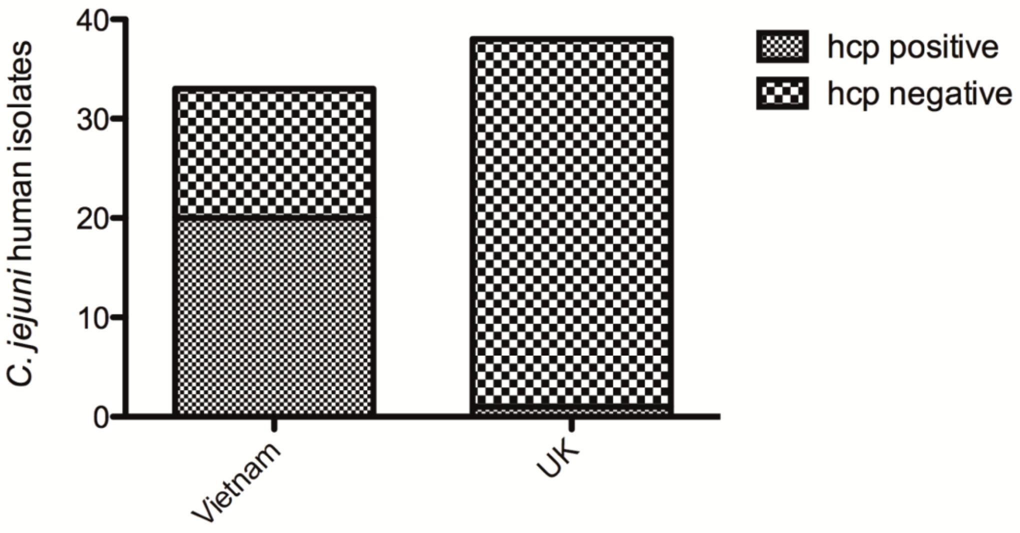

Technical Appendix Figure 2. Prevalence of T6SS genetic marker *hcp* in *Campylobacter jejuni* isolated from humans in Vietnam and the UK. Multiplex PCR was performed on genomic DNA purified from *C. jejuni* that were isolated from humans from Vietnam and the UK. Conserved T6SS gene *hcp* was used as a marker for a complete T6SS cluster. The conserved housekeeping gene *gltA* was used as a positive control.

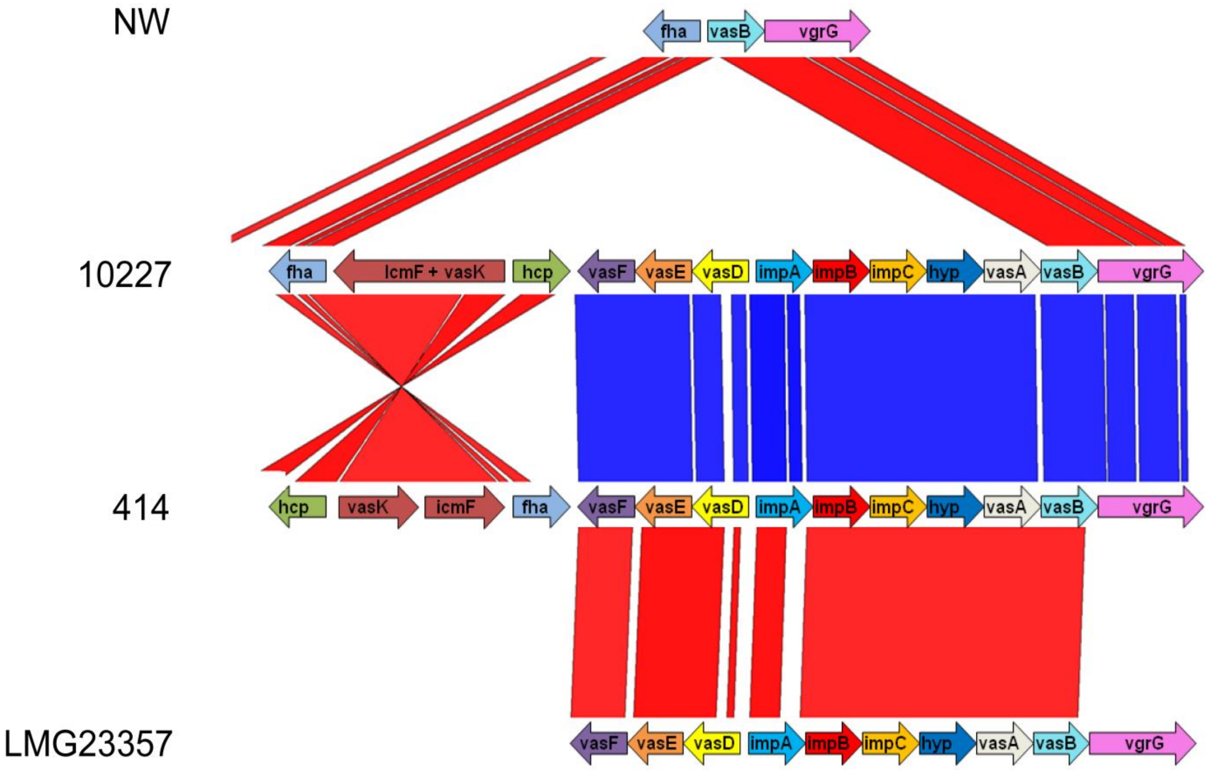

Technical Appendix Figure 3. Comparison of the gene orders in the T6SS gene clusters found in *Campylobacter jejuni*. The figure shows BLASTN alignments between representatives of each of the two gene-order types: strain 10227 shares gene order with P854, ATCC 33560, 2008 979, LMG 23263, 30318, Cj1, 1997 14, 51037, 51494, 1997 10, Cj5, LMG 23210, 10186, 255, 20176. Strain 414 has a unique gene arrangement. Strains NW and LMG23357 are examples of partial T6SS gene clusters (vgrG is shown as absent from LMG23357 but is present in the assembly on a different contig). Figure visualised using Artemis comparison tool.

References

1. Takamiya M, Ozen A, Rasmussen M, Alter T, Gilbert T, Ussery DW, et al. Genome sequences of two stress-tolerant *Campylobacter jejuni* poultry strains, 305 and DFVF1099. J Bacteriol. 2011a;193:5546–7. PubMed http://dx.doi.org/10.1128/JB.05753-11

2. Takamiya M, Ozen A, Rasmussen M, Alter T, Gilbert T, Ussery DW, et al. Genome Sequence of *Campylobacter jejuni* strain 327, a strain isolated from a turkey slaughterhouse. Stand Genomic Sci. 2011b;4:113–22. PubMed http://dx.doi.org/10.4056/sigs.1313504

3. Hepworth PJ, Ashelford KE, Hinds J, Gould K, Witney AA, Williams NJ, et al. Genomic variations define divergence of water/wildlife-associated *Campylobacter jejuni* niche specialists from

common clonal complexes. Environ Microbiol. 2011;13:1549–60. PubMed http://dx.doi.org/10.1111/j.1462-2920.2011.02461.x

4. Pearson BM, Gaskin DJH, Segers RPM, Wells JM, Nuijten PJM, van Vliet AHM. The complete genome sequence of *Campylobacter jejuni* strain 81116 (NCTC11828). J Bacteriol. 2007;189:8402–3. PubMed http://dx.doi.org/10.1128/JB.01404-07

5. Russell RG, Blaser MJ, Sarmiento JI, Fox J. Experimental *Campylobacter jejuni infection* in *Macaca nemestrina*. Infect Immun. 1989;57:1438–44. PubMed

6. Poly F, Read TD, Chen Y-H, Monteiro M, Serichantalergs O, Pootong P, et al. Characterization of two *Campylobacter jejuni* strains for use in volunteer experimental-infection studies. Infect Immun. 2008;76:5655–67. PubMed http://dx.doi.org/10.1128/IAI.00780-08

7. Poly F, Read T, Tribble DR, Baqar S, Lorenzo M, Guerry P. Genome Sequence of a Clinical Isolate of *Campylobacter jejuni*. Infect Immun. 2007;75:3425–33. PubMed http://dx.doi.org/10.1128/IAI.00050-07

8. Jerome JP, Klahn BD, Bell J, Barrick JE, Brown CT, Mansfield LS. Draft Genome Sequences of Two *Campylobacter jejuni* Clinical Isolates, NW and D2600. J Bacteriol. 2012;194:5707–8. PubMed http://dx.doi.org/10.1128/JB.01338-12

9. Burrough ER, Sahin O, Plummer PJ, Zhang Q, Yaeger MJ. Pathogenicity of an emergent, ovine abortifacient *Campylobacter jejuni* clone orally inoculated into pregnant guinea pigs. Am J Vet Res. 2009;70:1269–76. PubMed http://dx.doi.org/10.2460/ajvr.70.10.1269

10. Luo Y, Sahin O, Dai L, Sippy R, Wu Z, Zhang Q. Development of a Loop-Mediated Isothermal Amplification Assay for Rapid, Sensitive and Specific Detection of a *Campylobacter jejuni* Clone. J Vet Med Sci. 2012;74:591–6. PubMed http://dx.doi.org/10.1292/jvms.11-0462

11. Zhang M, Li Q, He L, Meng F, Gu Y, Zheng M, et al. Association Study Between an Outbreak of Guillain-Barre Syndrome in Jilin, China, and Preceding *Campylobacter jejuni* Infection. Foodborne Pathog Dis. 2010;7:913–9. PubMed http://dx.doi.org/10.1089/fpd.2009.0493

12. Friis C, Wassenaar TM, Javed M, Snipen L, Lagesen K, Hallin PF, et al. Genomic characterization of *Campylobacter jejuni* strain M1. PLoS ONE. 2010;5:e12253. PubMed http://dx.doi.org/10.1371/journal.pone.0012253

13. Gundogdu O, Bentley SD, Holden MT, Parkhill J, Dorrell N, Wren BW. Re-annotation and re-analysis of the *Campylobacter jejuni* NCTC11168 genome sequence. BMC Genomics. 2007;8:162. PubMed http://dx.doi.org/10.1186/1471-2164-8-162

14. Biggs PJ, Fearnhead P, Hotter G, Mohan V, Collins-Emerson J, Kwan E, et al. Whole-genome comparison of two *Campylobacter jejuni* isolates of the same sequence type reveals multiple loci of different ancestral lineage. PLoS ONE. 2011;6:e27121. PubMed http://dx.doi.org/10.1371/journal.pone.0027121

15. Fouts DE, Mongodin EF, Mandrell RE, Miller WG, Rasko D, Ravel J, et al. Major structural differences and novel potential virulence mechanisms from the genomes of multiple campylobacter species. PLoS Biol. 2005;3:e15. PubMed http://dx.doi.org/10.1371/journal.pbio.0030015

16. Cooper KK, Cooper M. a, Zuccolo, A., Law, B., & Joens, L. Complete genome sequence of *Campylobacter jejuni* strain S3. J Bacteriol. 2011;193:1491–2. PubMed http://dx.doi.org/10.1128/JB.01475-10
